# A quantitative study of acetic acid emissions from historical cellulose acetate at room conditions

**DOI:** 10.1038/s40494-025-01551-y

**Published:** 2025-02-22

**Authors:** M. Novak, J. Grau-Bové, T. Rijavec, I. Kraševec, I. Kralj-Cigić, C. De Stefani, C. Checkley-Scott, M. Strlič

**Affiliations:** 1 UCL Institute for Sustainable Heritage, University College London, Central House, 14 Upper Woburn Pl, WC1H 0NN London, UK; 2 Heritage Science Laboratory, Faculty of Chemistry and Chemical Technology, University of Ljubljana, Večna pot 113, 1000 Ljubljana, Slovenia; 3 London Metropolitan Archives, 40 Northampton Rd, EC1R 0HB London, UK; 4 Conservation by Design Ltd., 9 Newmarket Ct, Kingston, MK10 0AG Milton Keynes, UK

## Abstract

Historic objects made of cellulose acetate are potential sources of indoor pollution in heritage collections. As these objects degrade, volatile organic compounds, including acetic acid, are produced and emitted. High concentrations of acetic acid can increase the degradation rate of both organic and inorganic objects stored nearby. In this study, micro-invasive sampling and ion chromatography were used to quantify acetic acid emission rates from objects made of cellulose acetate, including 3D objects and photographic negatives. It was observed that some degrading objects emit acetic acid at high rates, even under standard environmental conditions (20 °C and 30% RH) that are prescribed for storage of objects made of cellulose acetate. The emission rate was found to be governed by the internal diffusion for thicker cellulose acetate objects and by the surface evaporation for thinner objects. In the diffusion-controlled mode of emission, the airflow did not accelerate acetic acid emissions. To compare the storage options for cellulose acetate objects, the emission rates of acetic acid were used as the inputs for models predicting the equilibrium acetic acid concentrations in various enclosures (archival box, surface-coated archival box, metal can, and display case). It was observed that surface-coated boxes contained higher concentrations of acetic acid than other enclosures, mainly due to their low air exchange rates and deposition velocities.

## Introduction

The investigation of the emissions of volatiles from a variety of materials has been gaining increased interest from scientists in recent decades^1–4^. This is especially notable in the case of volatile organic compounds, which are generally considered the main sources of indoor gas pollution^3,5^. Volatile organic compounds (VOCs) are a class of low-weight organic molecules that exhibit high volatility owing to their low boiling points at standard atmospheric pressure. Consequently, they easily evaporate from solids and liquids even at standard room temperatures^5,6^. A large number of sources of indoor pollution, such as wood, paints, and coatings, can cause cumulative effects and generate high concentrations of VOCs. In many cases, these concentrations are much higher than the concentrations of external pollutants such as SO_2_, NO_*x*_, and ozone^1,7^.

In the heritage context, there is a growing awareness that some VOCs, most notably organic acids, present a potential risk for heritage materials, both organic and inorganic^1,7^. Of all organic acids, acetic acid is considered especially problematic, as it has the potential to increase the degradation of several material types in heritage collections, due to acid’s corrosivity and emission rates. Cellulose, the main component of paper, can degrade faster in the presence of acetic acid^8^. Metals and glass objects are susceptible to corrosion if exposed to acetic acid vapours^9^. Natural history specimens based on calcareous materials also show physical signs of degradation, such as efflorescence, when in the presence of acetic acid^10^. For acid-sensitive objects, concentrations of less than <10 μg m^−3^ of acetic acid are recommended, while for general collections the value is between 100 and 700 μg m^−3^ or up to 2500 μg m^−3^ ^11–13^. In museums and archives, various sources of acetic acid can be found in storage and exhibition areas. The best-known emitters are modern semi-synthetic and synthetic materials as well as construction and storage materials^14,15^. Some storage enclosures are made of acidic paperboard, which can emit high quantities of organic acids. Woods used for the construction of display cases and boxes, such as oaks and medium-density fibreboards, are known to emit high concentrations of acetic acid^16–18^. Synthetic paints, coatings, and adhesives are also well-studied sources of acetic acid emissions^8,16,18,19^. The qualitative determination of acetic acid emissions from storage materials and synthetic polymers has been conducted by several research groups^15,20–22^. These studies mostly relied on non-destructive sampling using solid-phase microextraction and subsequent analysis using gas chromatography–mass spectrometry. However, quantitative analyses of acetic acid emissions from heritage collections have been scarce. A few researchers have developed semi-quantitative and quantitative methods to determine the emissions of acetic acid from a variety of materials found in heritage institutions, such as storage and construction materials (display cases, enclosures, and wood)^23–28^. Most of these studies focused on determining acetic acid concentrations in display cases and reached similar conclusions; display cases made of high-emitting woods, such as oaks, show higher concentrations of acetic acid (between 20 and 2000 μg m^−3^) than those found in galleries and storage rooms suited for general collections (5–500 μg m^−3^). This is a consequence of wood being an active source of acetic acid, as well as the low air exchange rates of the display cases, which slows the release of acetic acid from the case. Heritage objects can also be sources of acetic acid. As objects made of organic materials, such as paper and plastic, degrade, acetic acid can be emitted as a degradation product^1,8,29–31^. However, the quantification of acetic acid emissions from heritage objects has been less studied, even though their emissions could have a cumulative effect on acetic acid concentrations in indoor environments, along with emissions from storage and construction materials. Several authors have studied acetic acid emissions from paper materials and concluded that some aged and acidic papers emit high levels of acetic acid^32,33^. Some studies have focused on the effect of environmental conditions on emissions from heritage objects, such as papers and photographic prints. Penichon et al. studied the acetic acid emission rates from photographic prints and noted that the emissions were higher at high temperatures^34^. Smedemark et al. studied emissions from paper and photographic materials, as well as their dependence on environmental conditions^14,35^. The authors came to a similar conclusion, as they observed a decrease in acetic acid emission at lower temperatures and relative humidity (RH). Grøntoft et al. detected acetic acid emissions also from canvases^36^.

In recent years, conservation scientists have recognised the importance of studying acetic acid emissions from synthetic heritage materials such as cellulose acetate. Cellulose acetate, a type of semi-synthetic plastic, degrades through hydrolytic degradation^37–39^. During this reaction, acetyl groups in the polymer are substituted with hydroxyl groups, and acetic acid is released as a byproduct of the reaction. This acid acts as a reaction catalyst for hydrolytic deacetylation because it is highly soluble in cellulose acetate and contributes to chain-breaking. This causes an increase in the rate of degradation^40^. The formation of acetic acid molecules causes a distinctive vinegar odour that is often found in the presence of old cellulose acetate^38,41^. Therefore, this degradation process is known as vinegar syndrome^42^. Nevertheless, a detailed quantitative study of acetic acid emissions from cellulose acetate objects, as well as how these emissions are affected by environmental conditions and appropriate storage strategies for actively degrading cellulose acetate objects, have not yet been conducted. Therefore, the main aim of this study was to determine the emission rates of acetic acid under the standard museum environmental conditions recommended for objects made of cellulose acetate. In addition, the mechanisms that govern acetic acid emissions and storage implications for emitting objects were investigated. This knowledge can lead to an improved understanding of how standard museum environments influence acetic acid emissions. Consequently, this would advise preservation practices and lead to more appropriate storage of cellulose acetate, by detecting and isolating high-emitting cellulose acetate from the vicinity of other objects. In addition, quantification of the emission rates of acetic acid could become an important tool for monitoring the degradation of objects made of cellulose acetate.

## Methodology

### Cellulose acetate samples

The acetic acid emission rates from a variety of naturally aged cellulose acetate objects, including three-dimensional and photographic objects, presented in Table 1, were investigated. The objects were bought from second-hand shops and flea markets and are part of the historic plastic collection of the UCL Institute for Sustainable Heritage and Heritage Science Laboratory at the Faculty of Chemistry and Chemical Technology, University of Ljubljana. However, the storage history of these objects is unknown. The main criterion for choosing objects for the study was that they were naturally aged, as it was assumed that their emission rates would be comparable to those of naturally aged cellulose acetate objects in heritage collections. In addition, both three-dimensional and photographic objects were included to determine whether their different chemical compositions would cause variations in the detected emission rates of acetic acid. Detailed knowledge of the ages of objects and their storage history is unknown. Before emission testing, the samples were chemically characterised using Fourier-transformed infra-red spectroscopy to ensure that they were composed of cellulose acetate. Three pieces of each object were sampled for the emission measurements; their surface areas are listed in Table 1. Objects were cut into three rectangular-shaped samples using a scalpel or a handsaw so that the final weight of each sample was 0.5 g. The surface areas (*SA*) of the samples were calculated using Eq. (1):

1 $${SA}=2{lw}+2{lh}+2{hw}$$

where *l* is the length of the sample (m), *w* is the width of the sample (m), and *h* is the height of the sample (m). The sample dimensions were measured using a digital caliper.

**Table 1:** Historic cellulose acetate samples used in the emission study

Object	Origin	Description	Sample dimensions (*l* × *w* × *h*) (cm)	Surface area of a sample (m^2^)
P1	UCL Institute for Sustainable Heritage	Brown-black pendant	2 × 2 × 1.6	0.0021
P2	UCL Institute for Sustainable Heritage	Photographic black and white negative	5.4 × 4 × 0.05	0.0044
P3	UCL Institute for Sustainable Heritage	Blue pen holder	2.6 × 1.5 × 1	0.0016
P4	University of Ljubljana	Light brown hairpin	2 × 1 × 0.5	0.0007
P5	UCL Institute for Sustainable Heritage	White-yellow brush for clothes	2.7 × 1 × 0.5	0.0009
P6	University of Ljubljana	Brown hair tie	2.5 × 1 × 0.5	0.0008
P7	University of Ljubljana	Brown hair comb	3.6 × 1 × 0.2	0.0009

### ATR-FTIR spectroscopy

The Fourier-transformed infra-red spectra of the cellulose acetate objects were recorded using a Bruker ALPHA FTIR spectrometer (Billerica, MA, USA) in attenuated total reflection mode. The measurements were performed on samples cut with a surgical scalpel (2 × 2 mm) because the original samples were too large to ensure good contact with the ATR crystal. The spectra were recorded in the wavenumber range 4000–400 cm^−1^. An average of 32 scans, with a resolution of 4 cm^−1^ was taken. For each sample, spectra were recorded at three different locations to ensure repeatability of the measurement.

### Sampling of acetic acid

Acetic acid was sampled from the cellulose acetate samples in the active sampling mode using a thermal extractor M-CTE250TI (Markes International Ltd., Bridgend, UK), with a total volume of 114 cm^3^ for each chamber. For each object, measurements were performed in triplicate, and the fourth microchamber was empty and used as a blank. The samples (as reported in Table 1) were not modified to ensure that the detected emission rates were comparable to those found for intact objects in heritage collections. The samples were left inside the chamber overnight under a stream of pure nitrogen (99.99%; BOC, Woking, UK), with an airflow of 200 ml min^-1^, to equilibrate with the chamber environment and to achieve several air exchanges before sampling. The sampling was performed in the bulk emission mode at 20 °C and 30% RH to match the recommended environmental conditions for the storage of cellulose acetate objects^42^. The temperature and RH inside the microchambers were monitored throughout the experiment using i-buttons T/RH loggers DS1923 (Measurement Systems Ltd., Newbury, UK). The loggers had an accuracy of ±0.5 °C for temperature and ±5% for RH. The emitted acetic acid was sampled on charcoal sorbent tubes (part number 226-01, Anasorb CSC, SKC Ltd., Dorset, UK) positioned on the microchamber outlets. Several standards recommend this sorbent for the sampling of acetic acid, such as the OSHA/NIOSH PV2119 standard^43,44^. Chamber airflow was monitored throughout the sampling period using a digital airflow meter GFM17 (Aalborg, New York, USA). For airflow-dependent testing, sampling was performed at three different airflows: 100, 200, and 400 ml min^−1^. This corresponds to the air exchange rates of 52.6, 105, and 210.5 h^−1^. Depending on the airflow, the sampling cycle lasted eight hours at 100 ml min^−1^, four hours at 200 ml min^−1^, and two hours at 400 ml min^−1^, so that a total volume of 48 l of air was sampled. Between each sampling, the microchambers were heated to 120 °C and cleaned with distilled water. After sampling, the absorbed acid was analysed by ion chromatography.

### Sample preparation and ion chromatography

Prior to analysis, acetic acid was extracted from the charcoal sorbent following an adapted procedure from the one described by the PV2119 standard (OSHA, USA)^44^. The glass tube with the charcoal sorbent was scored with a glass cutter, and carefully broken into two pieces, and each segment of the sorbent was transferred to a separate 15 mL centrifuge tube. The acetic acid was extracted from the sorbent with 5 ml of 10 mM NaOH (Sigma-Aldrich, St. Louis, MO, USA). The tubes were vortexed for 20 s and shaken on a mechanical shaker for 30 min. Before the analysis, the extracted solution was filtered using nylon membrane filters with a 0.45 μm pore size (Chrom4, Suhl, Germany).

The analysis was performed using a Dionex ICS-5000 ion chromatograph (Thermo Fisher Scientific, Waltham, MA, USA) and an anion exchange Dionex IonPac AS11-HC (4 × 250 mm) column. A Dionex AERS 500 4 mm anion suppressor (Thermo Fisher Scientific, Waltham, MA, USA) was used at a current of 56 mA with a conductivity detector. The analysis conditions were previously published^7^. Briefly, the following linear gradient of solvent A (MQ) and B (100 mM NaOH) was used for elution: 0–6 min 5% B, 6–13 min increasing to 10% B, 13–16 min increasing to 40% B, 16–17 min holding at 40% B, 17–19 min decreasing to 5% B and holding until 21 min. The rest to 100% was A. A flow of 1.5 ml/min was used. The injection volume was 25 μL^24,44^. The samples were injected into the ion chromatograph in duplicates. Acetate standards were prepared using sodium acetate salts (Sigma-Aldrich, St. Louis, MO, USA) at concentrations ranging between 0.1 and 10 mg/L before each instrumental run. The detected concentrations of acetate ions were calculated to acetic acid as the molar ratio of sodium acetate and acetic acid is 1:1. The detection limit was determined based on the levels of sorbent tube blanks, as prescribed by the ICH guideline^45^. Four replicates of sorbent tube blanks have been analysed, so that the contribution and uncertainty of the response have been evaluated. A blank sorbent tube was included in each set of sampling, which was subtracted from the sample response. The limit of detection was determined as the 3.3*standard deviation of blanks difference between the sample and the blank response, which was equal to 0.14 mg/L acetic acid or 0.7 µg of acetic acid. The recovery of acetic acid from the charcoal sorbent tube was not tested; however, as noted in the PV2119 standard, the recovery of acetic acid from the Anasorb sorbent tubes is between 98% and 99%^44^.

### Acetic acid emission rate calculations

After quantifying the adsorbed acetic acid, the chamber air concentration *c*_*ch*_ (mg m^−3^), emission rate *ER* (μg h^−1^), and area-specific emission rate *SER*_*a*_ (μg m^−2^ h^−1^) of acetic acid were determined using Eqs. (2)–(4):

2 $${{c}}_{{{ch}}}=\frac{{{m}}_{{{{AA}}}}}{{V_{1}}}$$

3 $${ER}={{c}}_{{{ch}}}* {N}* V_{2}{}$$

4 $${{SER}}_{{{a}}}=\frac{{ER}}{{A}}$$

where *m*_*AA*_ is the mass of the adsorbed acetic acid (mg), *V*_*1*_ is the volume of the sampled air (m^3^), *N* is the air exchange rate (h^−1^), *V*_*2*_ is the volume of the emission chamber (m^3^), and *A* is the surface area of the sample (m^2^)^18,35^. The mass of adsorbed acetic acid was calculated from the acetic acid peak areas and calibration curves. Emission rates represent the amount of acetic acid emitted from the object per hour, whereas surface emission rates represent the amount of acetic acid emitted from the object per surface area per hour. Both the emission rates and area-specific emission rates of acetic acid from cellulose acetate samples are presented in this paper to simplify the comparison between the calculated values and those found in the published literature. Both the *ER* and *SER*_*a*_ were averaged from triplicate measurements for each sample, and the uncertainty was expressed as the standard deviation of the three measurements.

### Predictive modelling of acetic acid concentrations

The determined acetic acid emission values were used to predict acetic acid concentrations for several types of enclosures used to store cellulose acetate objects: an archival box, surface-coated archival box (Moistop wrapping), metal can, and display case. The developed model predicted the effect of acetic acid emitted from individual cellulose acetate objects on the equilibrium acetic acid concentration inside the enclosure. Acetic acid emissions from other sources such as building or storage materials, and other objects were not considered in this model. The initial acetic acid concentration in the enclosures was set to 0 mg m^−3^. The model did not consider the presence of an acetic acid absorber inside the enclosure. The walls were modelled as sinks of acetic acid, represented by the deposition velocity. The model does not assume that this mechanism involves absorption or adsorption. Another simplification is that the acetic acid was not desorbed from the wall. This last simplification is acceptable if the model is used to predict an equilibrium concentration, rather than how concentration changes dynamically over long periods of time.

The model is presented using Eqs. (5) and (6), as follows:

5 $$\frac{\delta {c}}{\delta {t}}=\frac{({{in}}-{{out}})}{{V}}$$

6 $$\frac{\delta {c}_{i}}{\delta t}={c}_{0}+\left(\frac{{{ER}}}{V}-{v}_{{{d}}}\,\ast {c}_{i}\ast \frac{A}{V}-N\,\ast {c}_{i}\right)$$

where *δc*/*δt* is the partial derivative of the change in acid concentration over time (mg m^−3^ h^−1^), “in” is the inflow of acetic acid (mg h^−1^), “out” is the outflow of acetic acid (mg h^−1^), *c*_0_ is the acid concentration at time 0 (mg m^−3^), *c*_*i*_ is the acid concentration at time *t* (mg m^−3^), *ER* is the emission rate (mg h^−1^), *V* is the volume of the enclosure (m^3^), *A* is the surface area of the enclosure (m^2^), *v*_d_ is the gravitation-driven deposition velocity of acetic acid on the enclosure surface (m h^−1^), and *N* is the air exchange rate of the enclosure (h^−1^).

This model is based on a mass balance conducted by the authors. The mass balance follows the principle that the accumulation is the sum of the inputs and outputs of acetic acid. This is illustrated in Eq. (5). The inputs are infiltration (N * c_out_, which is zero), and emission (ER/*V*), while the outputs are exfiltration (N * c_*i*_) and deposition (*v*_d_*c_*i*_ * A/*V*). Equation (6) is obtained when adding up all these mass flows. The model inputs for each storage scenario are listed in Table 2. The air exchange values^46,47^, as well as the deposition velocities of acetic acid for different materials (paperboard, metal, and glass), were obtained from the literature^36,48,49^. For deposition velocities, two values for each enclosure were used, describing both scenarios with minimum (*v*_d,min_) and maximum (*v*_d,max_) deposition of acetic acid on enclosure walls. These values present the effect of various deposition velocities available in the literature on the acetic acid concentrations.

**Table 2:** Model inputs for different storage environments

Enclosure type	Enclosure description	Dimensions (height × width × depth) (m)	Volume (m^3^)	Surface area (m^2^)	Air exchange (h^−1^)^46,47^	Deposition velocity, *v*_d_ (m h^−1^)^36,48,49^
Archival box	Clamshell-type storage box made of unbuffered paperboard	0.21 × 0.297 × 0.07^47^	0.004^47^	0.20	5.2^47^	*v*_d,min_ 0.0576^48^ *v*_d,max_ 0.342^36^
Surface-coated archival box	Clamshell-type storage box made of unbuffered paperboard, wrapped in one layer of Moistop barrier foil^a^	0.21 × 0.297 ×0.07^47^	0.004^47^	0.20	0.2^47^	*v*_d,min_ 0.0576^48^ *v*_d,max_ 0.342^36^
Metal can	35 mm film can made of steel (London Metropolitan Archives collection)	0.3 × 0.042	0.003	0.18	0.2^b^	*v*_d,min_ 0.18^49^ *v*_d,max_ 0.50^49^
Display case	Glass with wooden construction	3 × 3 × 1	9	30	0.05^46^	*v*_d.min_ 0.00036^36^ *v*_d,max_ 0.36^36^

^a^Moistop barrier foil (Conservation by Design, Milton Keynes, UK, SUMSPP5100).

^b^The AER value of metal can was approximated to be similar to the surface-coated archival box, due to the layer of aluminium present in the Moistop foil.

## Results and discussion

### Chemical characterisation

**Fig. 1:**
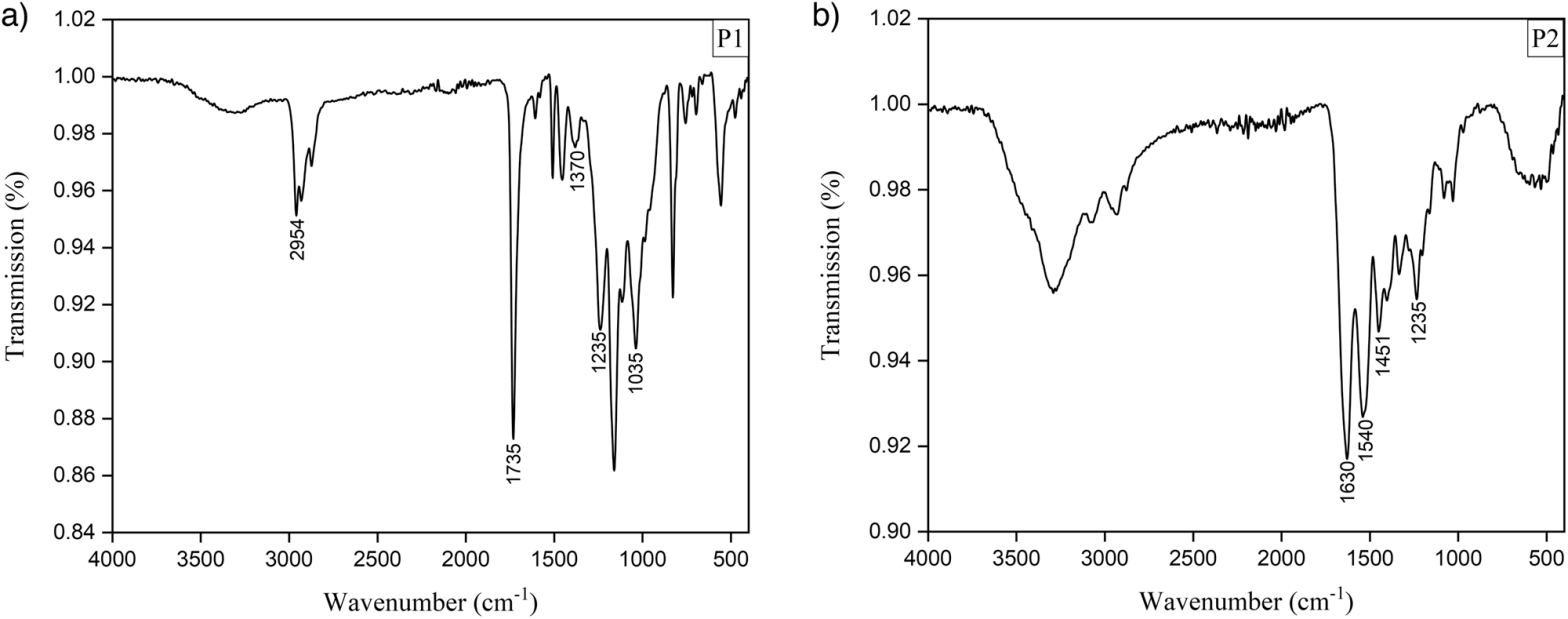
Chemical characterisation of the cellulose acetate samples. **a** FTIR spectrum of P1 (cellulose acetate) and **b** FTIR spectrum of P2 (gelatine-coated cellulose acetate).

The samples were analysed using ATR-FTIR spectroscopy to ensure that they were composed of cellulose acetate. Spectra of P1 and P2 are shown in Fig. 1. The band at 1735 cm^−1^ originated from C = O group stretching, the band at 1235 cm^−1^ from the ester group stretching, the band at ~1370 cm^−1^ from the –CH_3_ group bending and the band at 1035 cm^−1^ originated from a stretching of the C–O–C group^50^. The FTIR spectra for all samples indicate that the samples are made of cellulose acetate, except for sample P2, presented in Fig. 1b, whose spectrum shows different bands compared to other samples. As this sample is a photographic negative, its FTIR spectrum is dominated by the bands at 1630, 1540, and 1451 cm^−1^, which originate from the gelatine media present in photographic materials^51,52^. Although the gelatine layer was removed with a scalpel, and FTIR was taken from the plastic layer, there was a lack of the distinct cellulose acetate band at 1735 cm^−1^ was detected, however, the presence of bands at 2954, 1370, 1235, and 1035 cm^−1^ indicated cellulose acetate.

### Emission rates of acetic acid

**Fig. 2:**
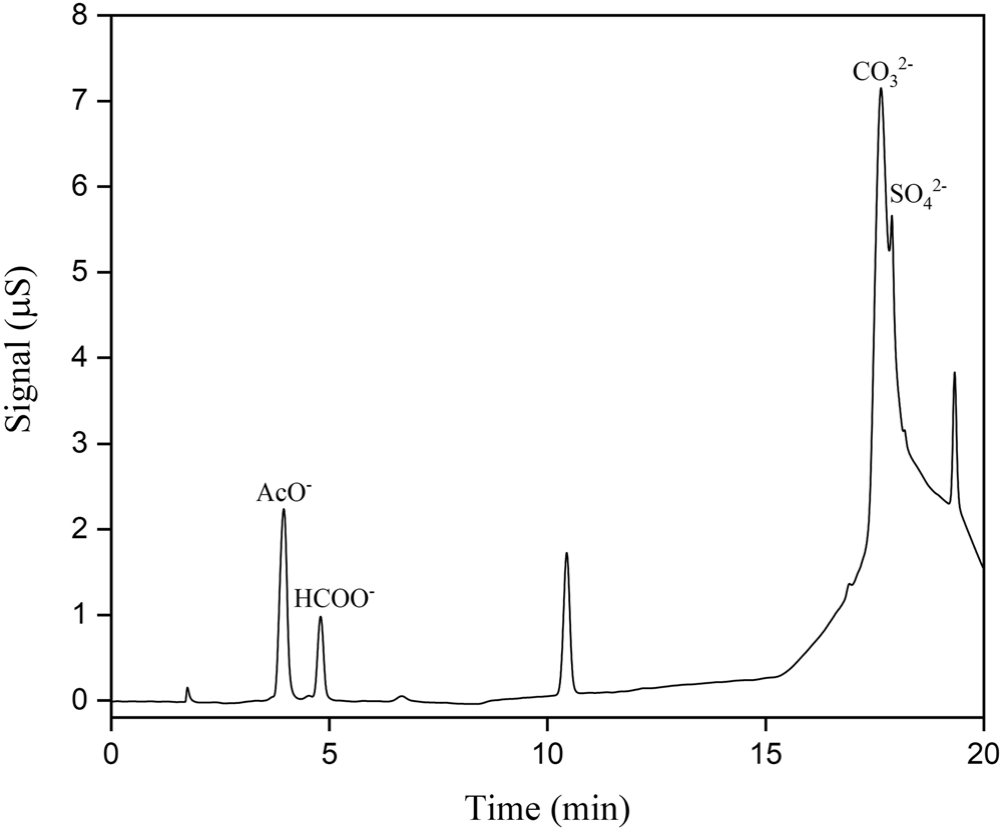
Ion chromatogram of sample P5 Acetic acid (AcO^−^) is retained at 3.97 min (emission sampling performed at 20 °C and 30% RH).

The chamber air concentrations and emission rates of acetic acid from seven historical cellulose acetate objects were quantified using a non-destructive sampling method and subsequent analysis with ion chromatography. An ion chromatogram of sample P5 is shown in Fig. 2. The detected chamber air concentrations of acetic acid were comparable for most samples, with approximate values between 20 and 30 μg m^−3^. The only exceptions are samples P1 and P5, which emit acetic acid at much higher rates, and show air chamber concentrations of (103.1 ± 2.8) and (393.3 ± 116.0) μg m^−3^, respectively. This result was not surprising, as both samples showed noticeable signs of degradation, such as a strong acidic smell. Although photographic negative P2 also showed signs of degradation in the form of surface channelling and an acidic smell, the detected acetic acid air concentrations were comparable to those of the less degraded samples. This can be attributed to the chemical composition of the samples or the emission of other acidic compounds released from the negative. Several researchers have noted that the gelatine layer in photographic negatives composed of cellulose acetate has a scavenging effect on acetic acid^38,53^. This effect is believed to stabilise the acid-catalysed degradation of cellulose acetate-based photographic negatives. This has the potential to lower acetic acid emissions from photographic negatives, as observed for P2.

**Fig. 3:**
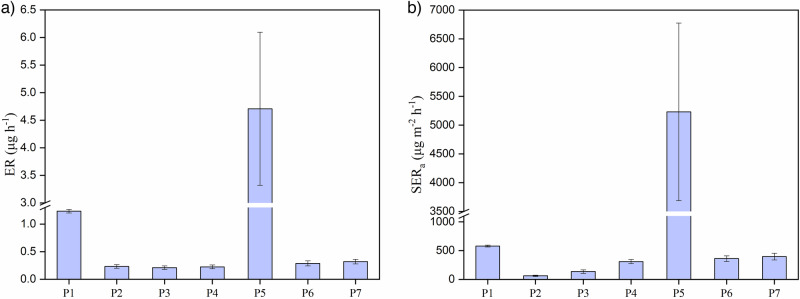
Determined acetic acid emission rates from the samples. **a** Emission rates of acetic acid and **b** area-specific emission rates of acetic acid. Measurements were performed at 20 °C and 30% RH. The error bars represent the standard deviations of the measurements, which were performed in triplicate for each sample.

The emission rates and area-specific emission rates were calculated from the chamber air concentrations using Eqs. (3) and (4); their values are presented in Fig. 3a, b, respectively. For the emission rates and area-specific emission rates, a comparable range of emission values was observed for most samples. The only exceptions were P1 and P5, which showed higher emission rates than those of the other samples. The average emission rates for the CA samples were as follows: (1.2 ± 0.03) μg h^−1^ for P1, (0.2 ± 0.03) μg h^−1^ for P2, (0.2 ± 0.03) μg h^−1^ for P3, (0.2 ± 0.03) μg h^−1^ for P4, (4.7 ± 1.4) μg h^−1^ for P5, (0.3 ± 0.04) μg h^−1^ for P6 and (0.3 ± 0.04) μg h^−1^ for P7. Regarding emissions from the surface area of the sample, sample P5 showed the highest value of (5230.4 ± 1542.9) μg m^−2^ h^−1^. The area-specific emission rates for other samples were (578.2 ± 15.5) μg m^−2^ h^−1^ for P1, (62.1 ± 2.1) μg m^−2^ h^−1^ for P2, (135.8 ± 29.3) μg m^−2^ h^−1^ for P3, (310.1 ± 35.3) μg m^−2^ h^−1^ for P4, (360.7 ± 42.35) μg m^−2^ h^−1^ for P6, and (396.1 ± 57.6) μg m^−2^ h^−1^ for P7.

A comparison of the acetic acid emissions of the samples showed that most tested samples showed similar emission rates, except for P1 and P5. As most of these objects, although historical, did not show any visible signs of degradation, lower acetic acid emissions were expected. The calculated emission rates were compared with literature values of acetic acid emissions from heritage objects. Although acetic acid emission rates have not been quantified for historical plastic objects yet, including cellulose acetate, several studies have focused on other heritage materials, such as paper, prints, wood, and packaging materials. Smedemark et al.^35^ researched area-specific acetic acid emissions from paper, photographs, and packaging materials and the effect of environmental conditions on these emissions. At 23 °C and 50% RH, paper emitted between 10 and 33 μg m^−2^ h^−1^ of acetic acid, and wood and packaging materials emitted between 50 and 300 μg m^−2^ h^−1^ of acetic acid. The photographic negative emission was 3185 μg m^−2^ h^−1^, which is comparable to the emissions detected in this study for sample P5, although this sample was a brush rather than a photographic negative. At 20% RH, similar to the RH used in this study, the authors noticed a decrease in the area-specific emission rates of the paper material, with reported values ranging from 6 to 123 μg m^−2^ h^−1^. These emission values were lower than those detected in this study, except for samples P2 and P3, whose values were comparable to these values. Another study conducted by the same authors concluded that aged papers had higher mass-specific emission rates than new papers^14^. At 23 °C and 50% RH, the highest value was 468 ng g^−1^ h^−1^, and under drier conditions, the emissions were lower (~10 ng g^−1^ h^−1^). Rhyl-Svendsen et al.^54^ measured the area-specific emission rates of acetic acid from wooden drawers and cases and calculated values in the range of 38.1–172.5 μg m^−2^ h^−1^ at 23 °C and 50% RH. A comparison of these values indicates that historic cellulose acetate objects can emit higher amounts of acetic acid than aged paper and wood under similar environmental conditions. Risholm-Sundman et al.^28^ tested the area-specific ER from different woods used to make display cases. The amount of acetic acid detected depended on the type of wood; birch emitted less than 10 μg m^−2^ h^−1^, whereas oak showed the highest value of 2800 μg m^−2^ h^−1^. However, this value is noticeably lower than that of P5. Another group investigated the area-specific emissions of acetic acid from canvas materials at 20 °C and 45% RH; the detected emissions were 219.6, 79.2, and 7.56 μg m^−2^ h^−1^, respectively^36^. Most of the cellulose acetate samples tested in this study showed much higher emission values, even though the emission testing was performed at lower RH. In conclusion, the detected emission rates for historical cellulose acetate samples at 20 °C and 30% RH show that this material can emit large quantities of acetic acid, even under the environmental conditions prescribed for this type of material. Therefore, the emission values presented herein provide quantitative evidence that cellulose acetate objects can be a major source of indoor pollution in heritage collections. As some environmental standards recommend higher airflows for objects made of cellulose acetate, as it is believed that air exchange removes excessive acetic acid from storage areas^42^, the effect of different airflows on acetic acid emissions was also studied in this research.

### The impact of airflows on the emission rates of acetic acid

**Fig. 4:**
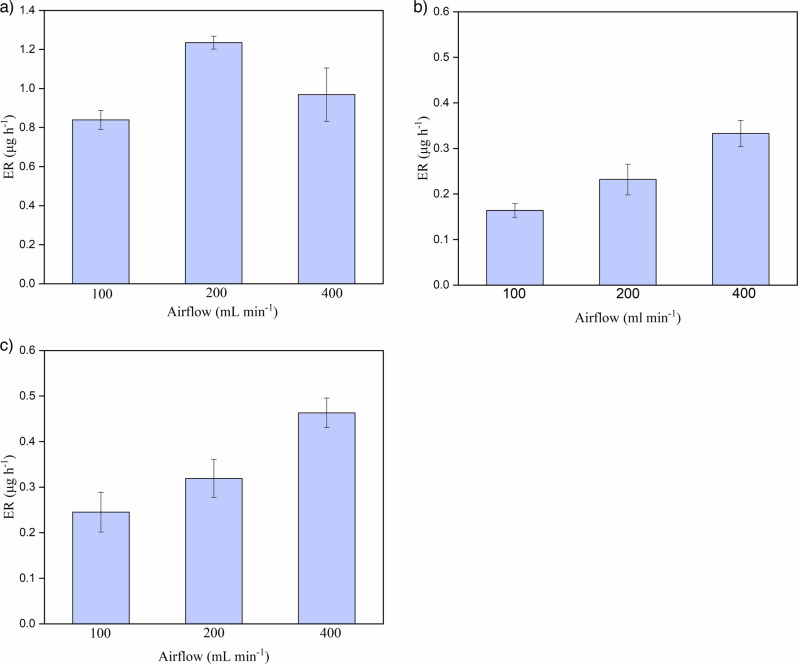
Effects of different airflows on the emission rates of acetic acid. **a** Sample P1, **b** sample P2, and **c** sample P7. Measurements were performed at 20 °C and 30% RH. The error bars represent the standard deviations of the measurements, which were performed in triplicate for each sample.

The effect of different airflows (100, 200, and 400 ml min^−1^) on the emission rates of acetic acid from aged cellulose acetate was tested. For samples P1, P3, P4, P5, and P6, the emission rates increased when the chamber airflow increased from 100 to 200 mL min^−1^. This observation is represented by sample P1, as shown in Fig. 4a. A gradual decline in the emission rates was observed for the highest airflow (400 ml min^−1^). According to the literature, the observed effect of airflow on emission rates could indicate a mechanism through which acetic acid emissions are controlled. These mechanisms include internal diffusion and surface evaporation. The emissions from a material can be controlled through one or both mechanisms at different times^55–57^. For materials where the change in airflow visibly influences its emissions, either by an observed increase or decrease, these emissions are governed by the surface evaporation mechanism^56,58,59^. This is a consequence of the effect of airflows on the mass transition coefficients at the material-air interface^60^. Higher airflows increase the mass transport coefficients and cause more rapid evaporation of acetic acid from the material surface. When airflows do not show a large influence, emissions are likely to be governed by the mechanism of internal diffusion.

This phenomenon is explained by the Biot number (Eq. (7)):

7 $${Bi}=\frac{{k}}{{D}}{L}$$

where *k* is the surface mass transfer coefficient, *D* is the diffusion coefficient, and *L* is the characteristic length (e.g. the thickness of a flat sample). For a constant thickness, when the Biot number is significantly smaller than 1, the transport in the bulk (diffusion) is higher than the transport at the surface (*k*), and therefore the system is evaporation-controlled. When the Biot number is higher than 1, the process is diffusion-controlled. Note that very small values of *L* could turn a diffusion-controlled system into an evaporation-controlled system.

The results for most of the studied samples (P1, P3, P4, P5, and P6) indicate that acetic acid emissions are governed by both surface evaporation and internal diffusion, depending on their properties and the measurement conditions. At low airflows (100 and 200 ml min^−1^), the emissions were governed by the evaporation of acetic acid accumulated on the surface, and the observed decline in emission rates at high airflows indicated a change to slower internal diffusion. Although the opposite is usual, the flow in the chamber at 400 ml min^−1^ could become more turbulent, which can affect the surface-air mass transfer to the degree emissions become diffusion-controlled. The surface evaporation of acetic acid is dependent on the mass transfer coefficient at the air-material interface, which in turn depends on the sample surface area, temperature, and airflow. The internal diffusion-governed emissions depend on the diffusion coefficients, which in turn depend on the environmental conditions of sampling, such as temperature and material properties, including porosity and thickness of the sample^59,61^. The influence of thickness (reported in Table 1 as value *h*) can be observed for samples P2 and P7, shown in Fig. 4b, c. For these two samples, an increase in the emissions at the highest airflow was observed. This result indicates that the acetic acid emissions from these two samples were controlled solely through surface evaporation at all the investigated airflow conditions. As the sampling was performed at one temperature (20 °C), the observed difference in the dominant mechanisms for these samples most probably comes from the variations in their physical properties, such as their thickness, compared to the other samples. As these two samples are thinner, their diffusion path is shorter, making it an evaporation-controlled mode of emission, as confirmed by the Biot equation. This means that for small values of *L*, objects may not be safe from the influence of airflow on surface evaporation. More research needs to be conducted on thin objects to ensure that they are not affected by airflow.

In conclusion, for the cellulose acetate objects used in this study, the effects of surface evaporation and internal diffusion on the acetic acid emission rates were observed for the majority of the studied samples, for the studied airflow range (100–400 ml min^−1^). Although this research focused on steady-state measurements of emissions, for time-dependent measurements, it is considered that for solid materials, surface evaporation controls emissions for shorter periods and internal diffusion governs emissions for longer periods^58,60^. However, this effect was not observed for the two samples in this study, namely P2 and P7, due to the effect of their thicknesses on acetic acid emissions. Notably, the observed differences in the dominant emission mechanisms occurred even though the microchambers used in this experiment were optimised to shorten the emission sampling times, according to the manufacturer. This is a probable consequence of the chambers’ high airflow.

### Prediction of equilibrium acetic acid concentrations in storage enclosures

**Fig. 5:**
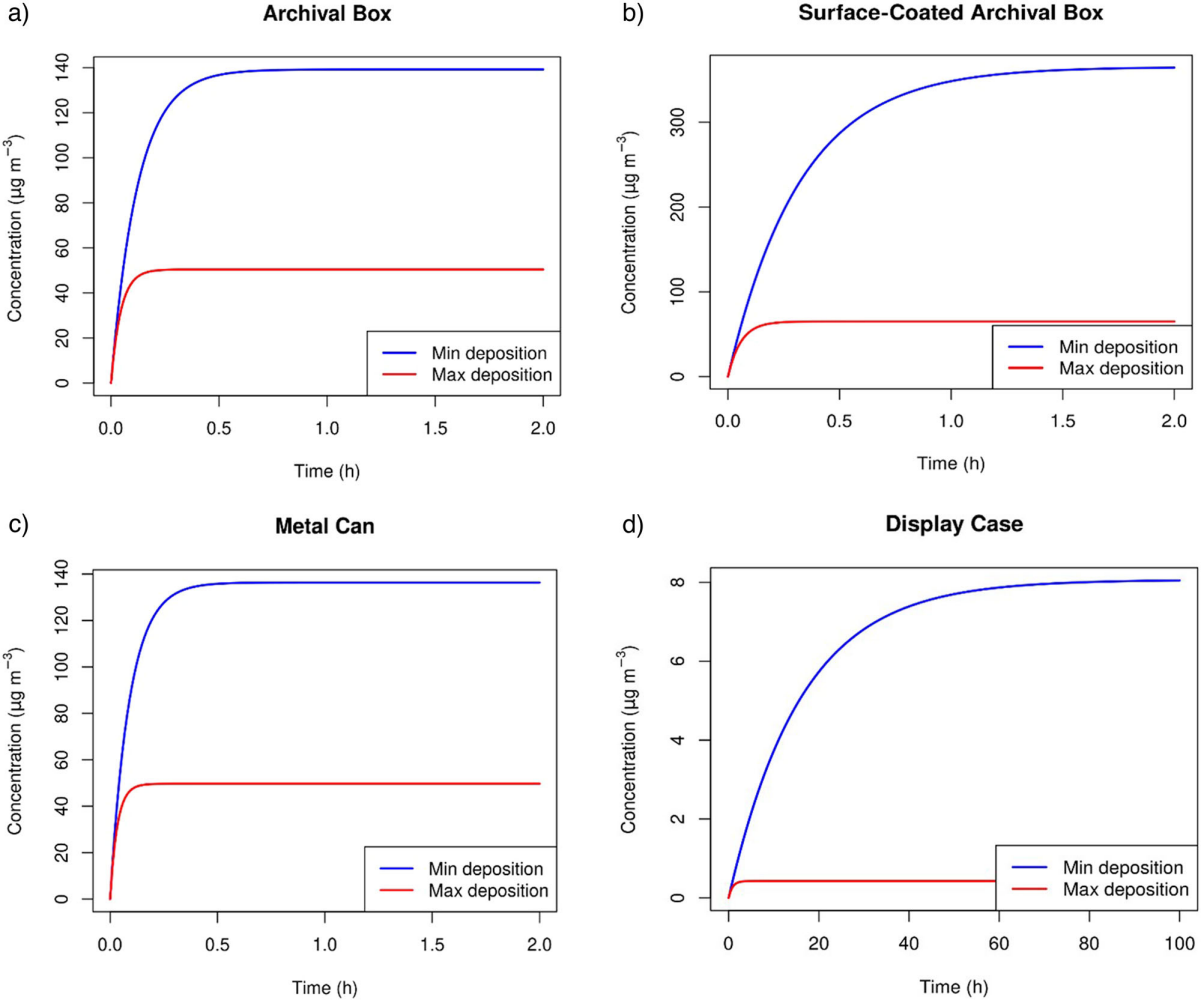
Predicted equilibrium acetic acid concentration emitted from sample P5. A scenario where the sample is stored in **a** archival box, **b** surface-coated archival box, **c** metalcan, and d display case.

The calculated emission rates of acetic acid from cellulose acetate samples were used to predict the acetic acid concentrations in the air as a consequence of these emissions under different storage scenarios. The effect of minimum and maximum acetic acid depositions on the acid concentrations inside an enclosure was also evaluated. Figure 5 and Table 3 present the predicted acetic acid concentrations emitted from an individual cellulose acetate sample under four different storage scenarios. The time dimension indicates the time point at which a cellulose acetate sample was placed inside an enclosure, and the enclosure was closed. As the plots for the storage scenarios of all tested samples showed a similar pattern, only the plots that represent sample P5 are shown.

**Table 3:** Predicted equilibrium acetic acid concentrations emitted from CA samples for different storage scenarios

Sample	Predicted *c*, archival box (μg m^−3^)		Predicted *c*, surface-coated archival box (μg m^−3^)		Predicted *c*, metal can (μg m^−3^)		Predicted *c*, display case (μg m^−3^)	
	*v*_d,min_	*v*_d,max_	*v*_d,min_	*v*_d,max_	*v*_d,min_	*v*_d,max_	*v*_d,min_	*v*_d,max_
P1	37.1	13.5	97.4	17.3	36.4	13.3	2.6	0.1
P2	7.1	2.6	18.7	3.3	7	2.5	0.5	0.02
P3	6.2	2.2	16.2	2.9	6.1	2.2	0.4	0.01
P4	6.8	2.5	17.9	3.2	6.6	2.4	0.5	0.02
P5	139.2	50.4	365.3	65.0	136.4	49.7	8.1	0.4
P6	8.9	3.3	23.5	4.2	8.8	3.2	0.6	0.03
P7	9.9	3.6	25.9	4.6	9.7	3.5	0.7	0.03

The predicted equilibrium concentrations of acetic acid were the highest for a scenario in which a sample was stored inside a surface-coated archival box with a minimum deposition. According to the literature, uncoated archival boxes made of paperboard have a permeable, porous structure and high air exchange values^47^. This low airtightness could be the reason why an uncoated archival box shows a lower equilibrium concentration of acetic acid compared to the surface-modified archival box. However, when compared to the display cases, the metal can and both the surface-modified and unmodified archival boxes showed higher equilibrium concentrations of acetic acid, for both minimum and maximum deposition velocities. One of the potential explanations for this effect is that owing to the larger internal volumes of display cases, a higher dilution of acetic acid occurs. This results in a lower acid concentration compared to small-volume enclosures, such as metal cans and archival boxes. From the model, it can be seen that both metal cans and archival boxes show comparable values of acetic acid concentrations. The comparable values between archival boxes and metal cans probably originate from their different deposition velocities and air exchange values. Although the maximum acid depositions of metal can (0.50 m h^−1^) are higher than the ones for the archival box (0.342 m h^−1^), both enclosures show similar values in acid concentration. This is due to the box having higher value of air exchange (5.2 h^−1^), compared to the metal can (0.2 h^−1^). According to Eq. (6), both higher values of air exchange and deposition velocities lower the concentration of acetic acid in enclosure headspace, and vice versa. To conclude, acetic acid concentration inside an enclosure increases in the following order: display case < metal can ~ archival box < surface-modified archival box. The odour thresholds for acetic acid vary and are reported between 14.7 and 2460 μg m^−3^, while the mild-irritative effect for humans is at 25 mg m^−3^ ^62^. Comparing these values to the predicted acetic acid concentrations for various enclosures, it can be concluded that display cases are below the minimum reported odour threshold values, while the surface-coated box surpasses minimum threshold values to some extent. Archival boxes and metal cans are between the two storage scenarios but can surpass the minimum odour values depending on the stored object and its emission rates.

In addition to variations in the equilibrium concentrations of acetic acid, the tested enclosures showed differences in the equilibration times. The display case with minimum acid deposition showed the slowest equilibration, with acetic acid equilibration taking ~4 days (Fig. 5). There are two reasons for this finding. The first was the low air exchange value of the display cases, which was 0.05 per hour. As display cases are made of glass material, acetic acid can only be released by air exchange through its openings, which is considered a slow process^63,64^. Second, the minimum deposition velocity of acetic acid onto the glass material was very low (0.00036 m h^−1^), which also slows the removal of acetic acid from the display case^36^. Archival box and metal can equilibrate in less than one hour; however, this process is more rapid in the case of maximum deposition of acetic acid. Similar observation was made for the surface-coated archival box. For minimum deposition of acetic acid, this enclosure equilibrates more slowly, in two hours, while for maximum deposition its equilibration takes less than one hour. These results indicate that the higher air exchange and deposition velocities also increase the rate of acetic acid removal from the enclosure headspace.

These predicted values shed new light on cellulose acetate storage, which has been a topic of debate for several decades, mainly in terms of identifying an optimal enclosure material that can protect cellulose acetate from moisture and acetic acid. Several studies that focused on the storage of cellulose acetate objects, mainly photographic materials, noted differences in object degradation states when stored in various enclosures. Some of these studies stated that cellulose acetate objects that had been stored in open and porous enclosures, such as archival boxes, were in a better state than those stored in metal and plastic cans, which showed visible signs of degradation^65,66^. The authors argued that one of the possible reasons for this is that archival boxes do not trap as much acetic acid as metal cans. Therefore, the object is less exposed to acetic acid, which is a hydrolytic degradation catalyst, and this stabilises the material in the long term. However, because these studies were performed at higher temperatures and RH, it was difficult to predict the material behaviour under mild conditions, such as room temperature. Consequently, enclosures made of metal and plastic are often preferred because of their low permeability to external moisture, which is the main reactant of the hydrolytic degradation of cellulose acetate^40,67^. In this study, the acetic acid emission measurements were performed at room temperature and low RH, and the results showed that enclosures such as metal cans do not seem to trap excessive amounts of acetic acid which could cause a rapid degradation of cellulose acetate objects. To conclude, proper storage strategies for modern plastic materials are still not fully understood and are a part of ongoing research. It is vital to protect objects from moisture, which initiates the degradation mechanism in cellulose acetate. However, for ageing objects, it is vital to remove acetic acid released from the vicinity of other objects to prevent their premature degradation. This is another practice that is often employed for cellulose acetate storage, and it relies on the use of activated carbons, zeolites, and, more recently, metal-organic frameworks to trap acetic acid that is released from the stored objects^68,69^. As mentioned earlier, the influence of pollutant absorbents was not included in this model. Similar to the observed effect of air exchange rates and deposition velocities on predicted equilibrium concentrations, the presence of an acid adsorbent would be another strategy for keeping the low concentrations of acetic acid in enclosures.

## Conclusions

In this study, the emission rates of acetic acid from selected historic cellulose acetate objects were determined using micro-destructive sampling and subsequent ion chromatography analysis. Although the samples were naturally aged, most showed comparable emission rates, which, according to the literature, were in the lower range of the acetic acid emission values. However, some samples that had observable signs of degradation, such as a strong acidic smell, showed higher emission rates. These results indicated that these samples were in an advanced state of degradation. Moreover, their acetic acid emission rates were higher than those of other types of heritage materials such as acidic paper and wood. Regarding the mechanism governing acetic acid emissions from cellulose acetate objects, it was concluded that internal diffusion was the dominant rate-determining step for most samples at airflow of 400 ml min^−1^; however, a surface evaporation-controlled mode of acetic acid emissions was observed for thinner samples, such as photographic negatives. For objects whose acetic acid emissions are governed by internal diffusion, increased air movement around the object does not influence its acetic acid emissions. This implies that some cellulose acetate objects can be stored in places with high air movement without the risk of any increased degradation of the material. At temperatures higher than the measured 20 °C, the evaporation constant is expected to increase, causing the diffusion-controlled mode to become predominant for even more objects. Acetic acid emission rates were used as model inputs to determine the acetic acid concentrations emitted from objects stored in four different enclosures. The predicted concentration values showed that some enclosures used to store cellulose acetate objects should contain high concentrations of acetic acid, which could cause increased degradation. For the four storage scenarios tested in this study, it was noted that some enclosures, such as surface-coated archival boxes, contained more accumulated acetic acid than the uncoated archival boxes and metal cans. This is a potential consequence of the lower air exchange rates and deposition velocities. In addition, display cases showed lower concentrations of acetic acid because of their larger volume, which diluted the emitted acetic acid more effectively than in the small-volume enclosures.

## Data Availability

No datasets were generated or analysed during the current study.

## Abbreviations

ATR: Attenuated total reflectance
AER: Air exchange rate
CA: Cellulose acetate
ER: Emission rates
EVA: Ethylene-vinyl acetate
FTIR: Fourier-transformed infra-red spectroscopy
RH: Relative humidity
SER_a_: Area-specific emission rates
T: Temperature
VOCs: Volatile organic compounds
